# Surgical Cases Occurring in the Mercy Hospital of Chicago

**Published:** 1858-04

**Authors:** J. W. Freer, H. A. Johnson

**Affiliations:** Surgeon; Surgeon


### ARTICLE III.

### SURGICAL CASES OCCURRING IN THE MERCY HOSPITAL OF CHICAGO, DURING THE COLLEGE SESSION OF 1856-7.

PROFS. J. W. FREER AND H. A. JOHNSON, SURGEONS.

REPORTED BY C. N. ELLINWOOD, ASS'T.

1. Case of Gun-shot Wound—Death. Care of Dr. Johnson.
2. Two cases of Ununited Fracture of the Femur. Treated by Dr. Brainard's method, successfully. Care of Dr. Freer.
3. Case of Fracture of the Tibia, ununited at eight weeks. Care of Dr. Johnson.
4. Case of Fracture of the Femur in a Boy. Speedy union. Care of Drs. Freer and Johnson.
5. Two cases of Varicose Veins. Treated by transfixing the Veins with the twisted suture. Care of Dr. Freer.

6. Case of Poplitœal Aneurism. Treated by ligature of the Femoral Artery. Recovery. Care of Dr. Freer.

7. Two cases of Phlegmonous Erysipelas. Care of Dr. Freer.

8. Case of Colloid Cancer, involving the Knee Joint. Amputation at the Thigh. Recovery. Care of Dr. Freer.

1. *Case of Gun-shot Wound, lacerating the soft parts and mangling the bones of the Fore Arm.*—Mike White was admitted into the hospital, Nov. 3d, at 9½ o'clock P. M. The accident occurred fourteen miles in the country the same afternoon at three o'clock. The immediate shock to the system was not severe. The hemorrhage was profuse. A ligature around the arm was soon applied, which in part suppressed it. He was brought to the city on a hand-car, and had drank freely of whisky during the time. On arriving at the hospital, the extremities were cold, pulse feeble and slow, and a prostration of the system. The hemorrhage was suppressed by compression. Efforts made to induce a reaction, preparatory to an operation, all of which failed, and at three o'clock the following morning he died.

2. *Ununited Fracture of the Femur.*—D. F. fractured his femur two inches below the trochanter major, very oblique, on the 1st of September.

*Dec. 17th.* No union had taken place; a shortening of two and a half inches; a curvature outwards, and a perfect mobility of the fractured extremities of the bone.

Dr. Brainard's new operation for the treatment of ununited fractures was performed with a small sized perforator. The perforations were much between the fractured surfaces, and in three directions, into the extremities of the bone. Splints retaining the limb in the straight position, and extension and counter-extension, were applied. A slight soreness of the part for three days was all the effect complained of by the patient.

*Jan. 3d.* Perforations repeated, as before, with a larger instrument.

*Jan. 29th.* Perforations repeated, between the fractured surfaces only.

*Feb. 10th.* The union of the bone has taken place. Extension taken off, and a starch bandage applied.

*Feb. 20th.* Able to get up on crutches.

*Feb. 25th.* Walks the ward comfortably. The shortening is not so great, as a high heel on the boot makes the limb long enough. The only difficulty remaining, is a stiffness of the knee joint, which is fast yielding.

3. *Fracture of the Tibia.*—At the expiration of eight weeks, the patient, Peter F. was admitted to hospital, November 10th, with no union of the bone. His general health was poor. He had been confined to a small room, a typhoid atmosphere, and poor diet. The flesh was flabby, and several abscesses had formed, one extending to the point of fracture, which were discharging pus. Subsultus tendinum well marked. He was put upon a good diet, one-twelfth of a grain of strychnine three times a day, and the limb placed in a fracture box. Stimulating injections into the abscesses; under which treatment he continued to improve.

*Dec. 22d.* But little discharge of pus, and a considerable callus thrown out around the point of fracture.

*Feb. 1st.* The patient is able to lift the limb around, and says it is quite strong.

*Feb. 15th.* Is able to get up and down stairs, and bear a considerable weight on the leg.

4. *Fracture of the Femur in a Boy—Speedy Recovery.*—Billy, aged five years, admitted September 24, with a fracture of the thigh, about the middle. The accident occurred the day previous, several miles in the country. The member was dressed in the straight position, with simple splints. In making the counter-extension, some difficulty was experienced from the perineal band and the urine, causing excoriations, to which an application of

℞ Glycerine, f ℥iij.
Soda bor., ʒi.
Tannin, gr. xv. M.; was made twice a-day.

*Nov. 1st.* Dressings removed, and the bone found to be united.

*Nov. 5th.* Able to walk; no perceptible shortening.

5. *Two cases of Varicose Veins, treated by transfixing the Vein with Twisted Sutures.*—These were cases of laboring men, whose employment had been with the shovel. No effect being derived from compression and the horizontal posture, the veins were enclosed within needles, and the circulation entirely cut off by means of a ligature, with a view to the obliteration of the vessel.

First case, of D. B. The internal saphena vein was operated on in this way, three needles being used, Dec. 2d. Dec. 13th. needles withdrawn, a considerable ulceration having occurred. Dec. 19th, discharged, cured.

Second case, of P. H. The same operation performed, Feb. 2d, with the same result.

6. *Case of Poplitœal Aneurism, treated by Ligature of the Femoral Artery—Recovery.*—M. D., admitted Dec. 21st. Compression had been applied before coming to the hospital without success. The operation of ligature of the femoral artery was performed, and the patient kept moderately under the influence of opiates.

*Jan. 15th.* Ligature came away, wound healed kindly, and no unpleasant symptoms follow the operation.

*Jan. 20th.* Discharged.

7. *Two cases of Plegmonous Erysipelas.*—T. G., admitted Nov. 21st. Erysipelas of foot, commenced from the chafing of a boot, three days before, and is extending up the leg, now nearly to the knee; pulse 96, compressible; constitutional symptoms severe.

℞ Quininæ sulph., gr. iij.; opii, gr. i., M., every two hours; and ℞ Iodine, gr. xx.; iodide potass., ʒss.; aqua, f ℥j.; M.; apply to entire diseased surface once a day.

*Nov. 23d.* Constitutional symptoms subsided; free discharge of pus.

*Dec. 1st.* Presents the appearance of deep ulcer. Stimulating ointments applied.

Thos. B., admitted Dec. 23d. Erysipelas, originating in a wound in the leg, by a sharp instrument, five days before entering the hospital. A large abscess had formed beneath the superficial muscles, which was freely opened, and a perfect coating of collodion put around the limb above the appearance of the disease, and for three days was given, ℞ Quinia, gr. iij., opii, gr. ½, every four hours.

*Jan. 5th.* Discharged, well.

8. *Case of Colloid Cancer, involving the Knee Joint—Amputation—Recovery.*—O. H., aged thirty-three years, admitted Feb. 7th, with malignant disease, involving the upper half of the tibia and the condyles of the femur. Amputation at the thigh performed, Feb. 9th. The operation was very well borne. The wound healed in part by the first intention.

*Feb. 17th.* A considerable discharge of sanies from the stump.

*Feb. 20th.* Ligatures came away. Stump almost entirely healed. No appearance of the return of the disease. Patient feels "very well."

